# Supplementary material for: Complexome profiling of the Chlamydomonas psb28 mutant reveals TEF5 as an early PSII assembly factor
Source: Plant Cell. 2025 Mar 17;37(6):koaf055. doi: 10.1093/plcell/koaf055 (PMC12164590; doi:10.1093/plcell/koaf055)
Supplement: koaf055_Supplementary_Data [file koaf055_supplementary_data.zip › psb28_interactive.zip!/plots/intensity/qualityPlots/peptides/CGLD25_Cre05.g234750.t1.2.html]

### Cre05.g234750.t1.2

Trivial name: CGLD25  
SeqCoverage [median]: 4.00  
Mass: 34.62 kDa  
  
 

| Sequence | MissedCleavage | Mass [Da] | wtIntensity | muIntensity | Score |
| --- | --- | --- | --- | --- | --- |
| ILGIGQK | 0 | 727.46 | 531.81 | 4166.49 | 94.95 |
| LFDESR | 0 | 765.37 | 0.00 | 1447.06 | 99.92 |
|  |  |  | 531.8 | 5613.5 |  |
